# Supplementary material for: Venom Variation of Neonate and Adult Chinese Cobras in Captivity concerning Their Foraging Strategies
Source: Toxins (Basel). 2022 Aug 29;14(9):598. doi: 10.3390/toxins14090598 (PMC9501182; doi:10.3390/toxins14090598)
Supplement: Supplementary file 1 [file toxins-14-00598-s001.zip › toxins-1845235-supplementary.pdf]

toxins

MDPI

# Venom Variation of Neonate and Adult Chinese Cobras in Captivity Concerning Their Foraging Strategies

**Xuekui Nie, Qianzi Chen, Chen Wang, Wangxiang Huang, Ren Lai, Qiumin Lu, Qiyi He and Xiaodong Yu**

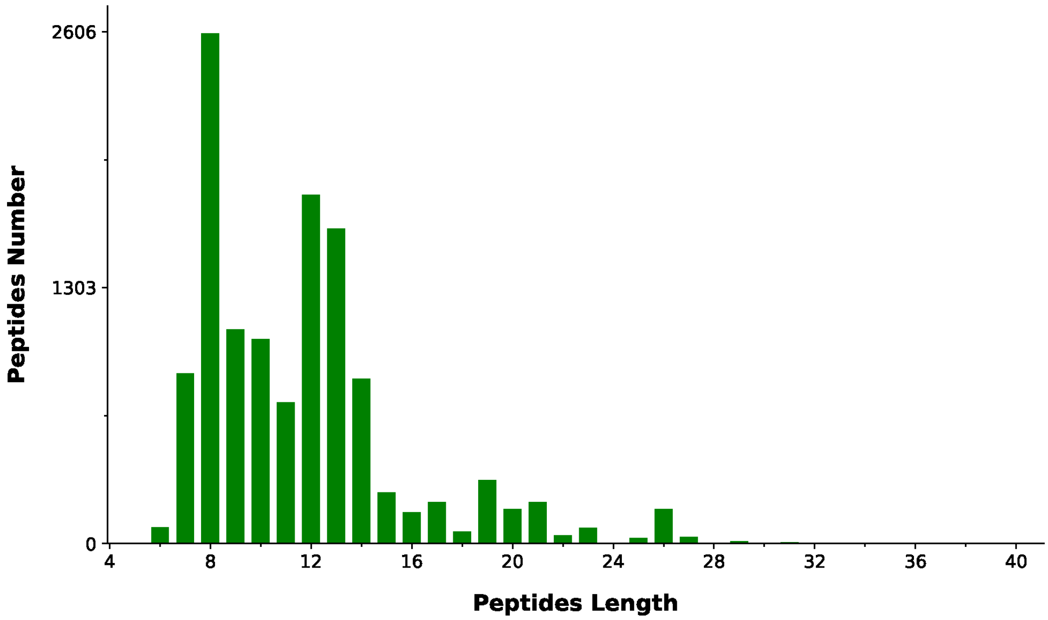


**Figure S1.** Distribution of peptides in enzymatic hydrolysis of *N. atra* snake venom.

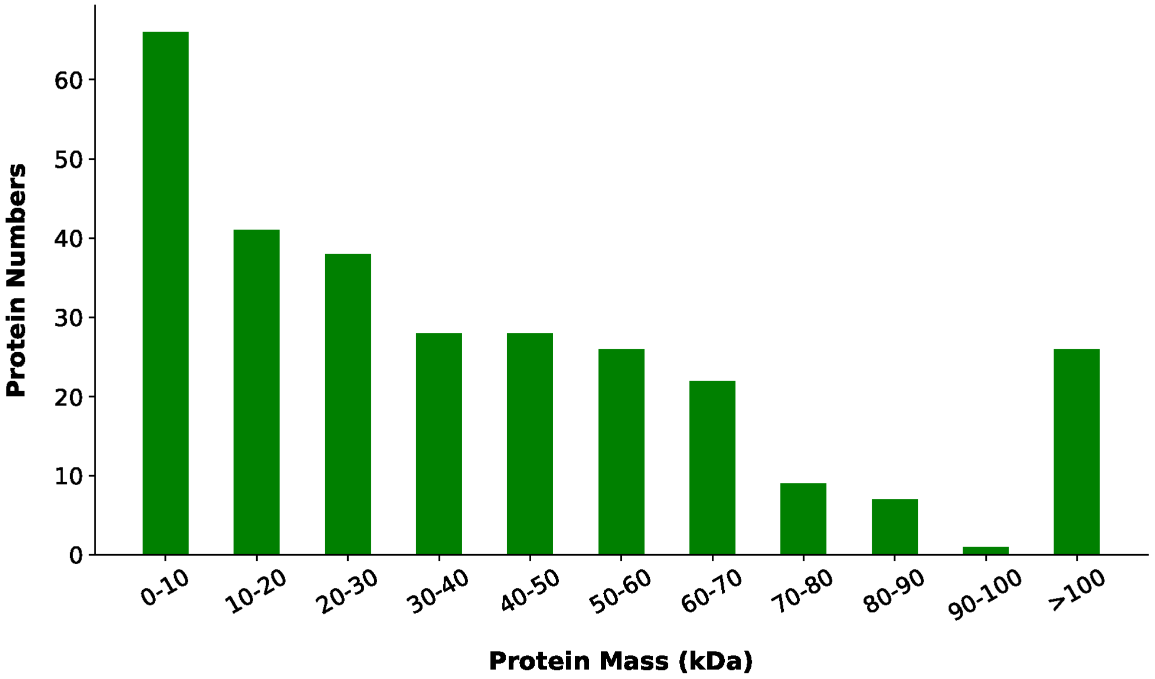


**Figure S2.** Molecular weight distribution of *N. atra* venom protein.

**Table S1.** Protein matches that were obtained by tandem MS/MS of tryptic peptides from captive *N. atra* venom in different ages and genders.

| Accession | Positions in Accession | Proteins | PSMs | q-value | Confidence | Theo. MH+ [Da] | Unique Matching Peptide Sequences | Protein Name | Species |
|---|---|---|---|---|---|---|---|---|---|
| V8N8G0 | 84-100 | 1 | 1 | 0.00085197 | High | 2346.2619 | [R].QVPDSAGTATAYLCGVK.[A] | Alkaline phosphatase | *O. hannah* |
| | 138-146 | 3 | 1 | 0.00923644 | High | 1237.74352 | [K].SVGIVTTTR.[V] | | |
| Q92035 | 101-108 | 3 | 1 | 0.00085197 | High | 1281.7486 | [K].NLYLDLAR.[C] | Acetylcholinesterase | *B. fasciatus* |
| | 354-382 | 2 | 2 | 0.00085197 | High | 3852.89944 | [R].MGVPHANDIATEAVVLQYTDWQDQDNGEK.[N] | | |
| | 247-263 | 2 | 2 | 0.00085197 | High | 2353.13481 | [K].QLGCHFNNDSELVSCLR.[S] | | |
| | 31-39 | 2 | 2 | 0.00085197 | High | 1321.75475 | [K].VATQTGWVR.[G] | | |
| | 414-422 | 2 | 27 | 0.00085197 | High | 1487.79661 | [K].VYAYLFDHR.[A] | | |
| | 264-273 | 2 | 5 | 0.00085197 | High | 2027.26808 | [R].SKTPQELIAK.[E] | | |
| | 266-273 | 2 | 5 | 0.00085197 | High | 1507.93395 | [K].TPQELIAK.[E] | | |
| | 475-484 | 1 | 1 | 0.00085197 | High | 1623.88337 | [R].TGNPTDPADK.[N] | | |

| | | | | | | | | | |
|---|---|---|---|---|---|---|---|---|---|
| | 385-409 | 2 | 1 | 0.00085197 | High | 3440.76517 | [R].EALDDIVGDHNVICPVVQFANDYTK.[R] | | |
| | 312-321 | 2 | 3 | 0.00085197 | High | 1708.08643 | [K].ETQLLLGVVK.[D] | | |
| | 274-281 | 2 | 17 | 0.00085197 | High | 1629.9496 | [K].EWSVLPYK.[S] | | |
| | 514-526 | 2 | 30 | 0.00085197 | High | 2240.22942 | [R].AQICAFWNHFLPK.[L] | | |
| | 322-337 | 2 | 2 | 0.00085197 | High | 2401.29352 | [K].DEGSYFLIYGLPGFSK.[D] | | |
| | 312-321 | 2 | 1 | 0.00454195 | High | 1403.87928 | [K].ETQLLLGVVK.[D] | | |
| | 264-273 | 2 | 1 | 0.0058016 | High | 1723.06094 | [R].SKTPQELIAK.[E] | | |
| Q9DF56 | 131-140 | 1 | 1 | 0.00085197 | High | 1748.96493 | [R].ICFAGAPYNK.[E] | Acidic phospholipase A2 | *O. hannah* |
| P00598 | 122-142 | 4 | 5 | 0.00085197 | High | 2964.55333 | [R].LAAICFAGAPYNNNNYNIDLK.[A] | Acidic phospholipase A2 | *N. atra* |
| | 122-142 | 4 | 2 | 0.00085197 | High | 2356.13904 | [R].LAAICFAGAPYNNNNYNIDLK.[A] | | |
| | 122-142 | 4 | 12 | 0.00085197 | High | 2660.34619 | [R].LAAICFAGAPYNNNNYNIDLK.[A] | | |
| Q9DF33 | 94-107 | 1 | 3 | 0.00454195 | High | 2043.94349 | [K].RYSYDCSEGTLTCK.[A] | Acidic phospholipase A2 | *O. hannah* |
| | 33-55 | 2 | 1 | 0.00454195 | High | 3876.88987 | [R].CCQVHDNCYNKAEKISGCWPYFK.[T] | Acidic phospholipase A2 | |
| P60045 | 73-86 | 1 | 2 | 0.00085197 | High | 2320.14449 | [K].TYSYECTQGTLTCK.[G] | Acidic phospholipase A2 3 | *N. sagittifera* |
| | 1-13 | 1 | 1 | 0.00085197 | High | 2216.25055 | [-].SNRPMPLNLYQFK.[N] | | |
| | 102-122 | 1 | 1 | 0.00085197 | High | 2922.53153 | [R].LAAICFAGAPYNDANYNIDLK.[A] | | |
| Q6T179 | 73-86 | 1 | 1 | 0.00085197 | High | 2320.14449 | [K].TYTYECSQGTLTCK.[G] | Acidic phospholipase A2 4 | *N. sagittifera* |
| | 1-13 | 1 | 1 | 0.00085197 | High | 1912.0434 | [-].SNRPMPLNIYQFK.[N] | | |
| | 73-86 | 1 | 1 | 0.00454195 | High | 2015.93734 | [K].TYTYECSQGTLTCK.[G] | | |
| Q9I900 | 107-121 | 1 | 2 | 0.00085197 | High | 1915.8016 | [K].GGNDACAAAVCDCDR.[L] | Acidic phospholipase A2 D | *N. sputatrix* |
| | 70-83 | 4 | 1 | 0.0032467 | High | 2378.0819 | [R].CCQVHDNCYGEAEK.[LI] | | |
| P25498 | 31-56 | 1 | 1 | 0.00454195 | High | 3546.62917 | [R].GGSGTPVDDLDRCCQIHDNCYNEAGK.[I] | Acidic phospholipase A2 E | *N. oxiana* |
| A4FS04 | 1-16 | 1 | 1 | 0.00085197 | High | 2303.19596 | [-].NLYQFKNMIQCTVPSR.[S] | Acidic phospholipase A2 natratoxin | *N. atra* |

| | | | | | | | | | |
|---|---|---|---|---|---|---|---|---|---|
| | 1-16 | 1 | 2 | 0.00454195 | High | 2361.20144 | [-].NLYQFKNMIQCTVPSR.[S] | | |
| P86542 | 7-15 | 1 | 4 | 0.0058016 | High | 1353.68257 | [K].NMIQCTVPS.[-] | Phospholipase A2 3 | *N. naja* |
| V8ND68 | 114-122 | 1 | 1 | 0.00085197 | High | 1799.96844 | [K].NNKDDPFWR.[H] | Phospholipase B-like | *O. hannah* |
| | 114-122 | 1 | 1 | 0.00454195 | High | 1495.76129 | [K].NNKDDPFWR.[H] | | |
| | 97-103 | 1 | 1 | 0.00454195 | High | 1808.09203 | [K].VKDFMQK.[Q] | | |
| Q7LZI1 | 47-55 | 2 | 1 | 0.00085197 | High | 1406.67274 | [K].NCFSSSLCK.[L] | Phospholipase A2 inhibitor | *N. kaouthia* |
| | 170-177 | 2 | 1 | 0.00085197 | High | 1529.8489 | [K].VECTDAVK.[I] | | |
| | 159-169 | 1 | 1 | 0.00085197 | High | 1962.12639 | [R].THEVDRNELIK.[V] | | |
| | 159-169 | 1 | 1 | 0.00085197 | High | 1657.91925 | [R].THEVDRNELIK.[V] | | |
| | 133-144 | 2 | 2 | 0.00085197 | High | 2022.08617 | [K].EMYPGDIAYNIK.[G] | | |
| | 133-144 | 2 | 2 | 0.00085197 | High | 2038.08108 | [K].EMYPGDIAYNIK.[G] | | |
| | 124-131 | 2 | 1 | 0.00165734 | High | 1270.68971 | [K].CINIAGYR.[K] | | |
| A0A6J1V2V7 | 119-138 | 1 | 1 | 0.00085197 | High | 2630.33421 | [R].DSPTFTNWAAGEPNTAGGTK.[Q] | PLIalpha-like protein | *N. scutatus* |
| V8NQ76 | 137-146 | 1 | 1 | 0.00085197 | High | 1669.92523 | [K].DSNDIEAVAK.[A] | Atriopeptidase | *O. hannah* |
| P00599 | 50-79 | 1 | 1 | 0.00165734 | High | 4624.23245 | [K].CYDEAEKISGCWPYIKTYTYESCQGTLTCK.[D] | Basic phospholipase A2 1 | *N. melanoleuca* |
| P60043 | 73-101 | 1 | 2 | 0.00085197 | High | 3598.49322 | [K].TYSYECKAGTLSCSGSNNSCAATVCDCDR.[L] | Basic phospholipase A2 1 | *N. sagittifera* |
| V8N495 | 67-73 | 2 | 1 | 0.00085197 | High | 1428.90701 | [R].VNYLALK.[E] | Carboxypeptidase | *O. hannah* |
| A0A6J1VS44 | 117-129 | 2 | 1 | 0.00085197 | High | 1716.90222 | [K].LVANMHGDETLGR.[Q] | Carboxypeptidase D | *N. scutatus* |
| I2C090 | 501-510 | 3 | 5 | 0.00085197 | High | 1462.85888 | [R].ITPDLIPSFR.[F] | O. venom factor | *O. hannah* |
| | 283-296 | 2 | 2 | 0.00085197 | High | 2106.24142 | [R].IPILDGDGEATLKR.[D] | | |
| Q91132 | 73-99 | 1 | 1 | 0.00085197 | High | 3132.624 | [R].VDMNPAGGMLVTPTIEIPAKEVSTDSR.[Q] | Cobra venom factor | *N. kaouthia* |
| | 1558-1567 | 1 | 1 | 0.00085197 | High | 1535.86134 | [R].AKTHQYISQR.[K] | | |
| | 936-946 | 1 | 24 | 0.00085197 | High | 1709.04529 | [K].GVGGTQLEVIK.[A] | | |

| | | | | | | | | | |
|---|---|---|---|---|---|---|---|---|---|
| | 398-414 | 1 | 27 | 0.00085197 | High | 2179.34974 | [K].LILNIPLNAQSLPITVR.[T] | | |
| | 468-477 | 1 | 1 | 0.00085197 | High | 1985.23237 | [K].GNANSLKQIK.[Y] | | |
| | 923-929 | 1 | 8 | 0.00165734 | High | 1367.91176 | [K].SIVTIVK.[L] | | |
| | 468-474 | 1 | 1 | 0.00454195 | High | 1311.78762 | [K].GNANSLK.[Q] | | |
| ENSNNAP00000012520.1 | 73-99 | 2 | 5 | 0.00085197 | High | 2814.36482 | [R].VDMNPAGGMLVTPTIEIPANEVSTDSR.[Q] | Cobra venom factor | *N. kaouthia* |
| | 73-99 | 2 | 3 | 0.00085197 | High | 2830.35973 | [R].VDMNPAGGMLVTPTIEIPANEVSTDSR.[Q] | | |
| | 73-99 | 2 | 2 | 0.00085197 | High | 3134.56688 | [R].VDMNPAGGMLVTPTIEIPANEVSTDSR.[Q] | | |
| | 73-99 | 2 | 4 | 0.00085197 | High | 3150.56179 | [R].VDMNPAGGMLVTPTIEIPANEVSTDSR.[Q] | | |
| | 390-397 | 2 | 1 | 0.00085197 | High | 1303.7078 | [R].TNHEVFPR.[E] | | |
| ENSNNAP00000021869.1 | 377-388 | 1 | 4 | 0.00085197 | High | 1812.94915 | [R].SDFPESWLWLTK.[D] | Cobra venom factor | *Naja kaouthia* |
| | 297-303 | 3 | 1 | 0.0058016 | High | 1225.64962 | [K].FQDQDLR.[K] | | |
| ENSNNAP00000023282.1 | 361-372 | 1 | 10 | 0.00085197 | High | 1612.85017 | [R].DGFIADSDIISR.[S] | Cobra venom factor | *N. kaouthia* |
| ENSNNAP00000010931.1 | 230-237 | 1 | 1 | 0.00085197 | High | 1552.89387 | [R].NDQNVVQK.[F] | Complement C3 | *N. naja* |
| | 161-176 | 1 | 5 | 0.00085197 | High | 2509.26369 | [K].LNSGAWNNYEQTTMQK.[M] | | |
| | 161-176 | 1 | 4 | 0.00085197 | High | 2493.26878 | [K].LNSGAWNNYEQTTMQK.[M] | | |
| | 161-176 | 1 | 1 | 0.00085197 | High | 2205.05655 | [K].LNSGAWNNYEQTTMQK.[M] | | |
| | 161-176 | 1 | 2 | 0.00085197 | High | 2189.06163 | [K].LNSGAWNNYEQTTMQK.[M] | | |
| | 70-82 | 1 | 6 | 0.00085197 | High | 2079.17703 | [R].IPVYSAYVYNPGK.[A] | | |
| | 202-222 | 2 | 2 | 0.00085197 | High | 2837.43708 | [R].VNRPSHLWSAACCLIDNNHLR.[S] | | |
| | 202-222 | 2 | 1 | 0.00085197 | High | 2533.22994 | [R].VNRPSHLWSAACCLIDNNHLR.[S] | | |

| | | | | | | | | | |
|---|---|---|---|---|---|---|---|---|---|
| | 146-160 | 3 | 5 | 0.00085197 | High | 2000.13874 | [K].SSTFTLTNIVPQFIK.[L] | | |
| | 146-160 | 3 | 7 | 0.00085197 | High | 2304.34589 | [K].SSTFTLTNIVPQFIK.[L] | | |
| | 36-49 | 1 | 39 | 0.00085197 | High | 1749.02299 | [R].ETPPVIGVPPSPVR.[I] | | |
| | 238-246 | 1 | 12 | 0.00085197 | High | 1672.97654 | [K].FTLGQLEEK.[L] | | |
| | 60-66 | 2 | 4 | 0.00085197 | High | 1465.85464 | [R].FATLYDK.[HQ] | | |
| | 247-265 | 2 | 2 | 0.00085197 | High | 2574.32064 | [K].LAQLYNVNHVSLFHSDCPR.[Q] | | |
| | 180-199 | 2 | 8 | 0.00085197 | High | 2414.24574 | [R].GCQQTFAVVGAVPGDTYIAR.[G] | | |
| | 131-145 | 2 | 2 | 0.00085197 | High | 2243.18128 | [R].GHLNPNGHQPDYSAK.[S] | | |
| | 36-49 | 1 | 1 | 0.00165734 | High | 1444.81584 | [R].ETPPVIGVPPSPVR.[I] | | |
| | 223-229 | 2 | 3 | 0.0058016 | High | 1120.67979 | [R].SWAILAR.[N] | | |
| ENSNNAP00000013063.1 | 562-570 | 1 | 3 | 0.00085197 | High | 1554.89916 | [R].IQMPGAAMK.[I] | Complement C3 | *N. naja* |
| | 259-270 | 1 | 7 | 0.00085197 | High | 1873.14428 | [K].VEGVAFVLFGVK.[I] | | |
| | 259-270 | 1 | 1 | 0.00085197 | High | 1568.93713 | [K].VEGVAFVLFGVK.[I] | | |
| Q01833 | 854-871 | 2 | 3 | 0.00085197 | High | 2346.17191 | [R].VELIYNPAFCSASTEGQR.[Y] | Complement C3 | *N. naja* |
| | 400-416 | 2 | 1 | 0.00085197 | High | 2209.3603 | [K].LILNTPLNIQSLPITVR.[T] | | |
| Q90WI6 | 125-134 | 1 | 2 | 0.00085197 | High | 1860.01809 | [K].NNEFCVEIVK.[F] | C-type lectin BML-1 | *B. multicinctus* |
| | 125-134 | 1 | 1 | 0.00085197 | High | 1555.81094 | [K].NNEFCVEIVK.[F] | | |
| | 100-107 | 1 | 8 | 0.00085197 | High | 1472.74933 | [K].YIWEWTDR.[S] | | |
| | 108-116 | 1 | 2 | 0.00085197 | High | 2065.22622 | [R].SKTDFLQWK.[K] | | |
| | 110-116 | 1 | 6 | 0.00085197 | High | 1545.89208 | [K].TDFLQWK.[K] | | |
| E3P6P4 | 44-55 | 2 | 9 | 0.00085197 | High | 1614.85592 | [K].AAAFAVQEYNAR.[S] | Cystatin | *N. kaouthia* |
| | 56-63 | 2 | 1 | 0.00085197 | High | 1561.86185 | [R].SANAHYYK.[E] | | |
| | 67-79 | 2 | 5 | 0.00085197 | High | 1952.13081 | [R].VVEAQSQVVAGEK.[Y] | | |
| | 103-112 | 1 | 9 | 0.00085197 | High | 1836.01809 | [K].EIQNCELPPK.[A] | | |

| | | | | | | | | | |
|---|---|---|---|---|---|---|---|---|---|
| | 80-88 | 2 | 2 | 0.00165734 | High | 1814.00877 | [K].YYLMMELVK.[T] | | |
| P86543 | 1-10 | 5 | 24 | 0.00085197 | High | 1472.73005 | [-].NVDFNSESTR.[R] | Cysteine-rich venom protein | *N. naja* |
| | 1-10 | 5 | 3 | 0.00085197 | High | 1168.52291 | [-].NVDFNSESTR.[R] | | |
| | 1-11 | 5 | 12 | 0.00085197 | High | 1628.83116 | [-].NVDFNSESTRR.[K] | | |
| P84807 | 1-11 | 1 | 5 | 0.00085197 | High | 1629.81518 | [-].DVDFNSESTRR.[K] | Cysteine-rich venom protein 25-A | *N. haje haje* |
| | 1-10 | 1 | 2 | 0.00165734 | High | 1473.71407 | [-].DVDFNSESTR.[R] | | |
| P84808 | 215-224 | 2 | 5 | 0.00085197 | High | 1919.95992 | [K].QNACQTEWMK.[S] | Cysteine-rich venom protein kaouthin-2 | *N. kaouthia* |
| | 215-224 | 2 | 16 | 0.00085197 | High | 1903.96501 | [K].QNACQTEWMK.[S] | | |
| | 215-224 | 2 | 6 | 0.00085197 | High | 1599.75786 | [K].QNACQTEWMK.[S] | | |
| | 158-179 | 3 | 2 | 0.00085197 | High | 3159.65027 | [K].YLYVCQYCPAGNIIGSIATPYK.[S] | | |
| | 158-179 | 3 | 1 | 0.00085197 | High | 2855.44312 | [K].YLYVCQYCPAGNIIGSIATPYK.[S] | | |
| | 227-234 | 2 | 5 | 0.00085197 | High | 1335.59271 | [K].CAASCFCR.[T] | | |
| | 202-214 | 2 | 6 | 0.00085197 | High | 2150.14206 | [K].HHNVFSNCQSLAK.[Q] | | |
| | 202-214 | 2 | 5 | 0.00085197 | High | 1845.93492 | [K].HHNVFSNCQSLAK.[Q] | | |
| | 90-107 | 4 | 1 | 0.00085197 | High | 2423.14432 | [K].IGCGENLFMSSQPYAWSR.[V] | | |
| | 90-107 | 4 | 4 | 0.00085197 | High | 2407.1494 | [K].IGCGENLFMSSQPYAWSR.[V] | | |
| | 90-107 | 4 | 1 | 0.00085197 | High | 2118.93717 | [K].IGCGENLFMSSQPYAWSR.[V] | | |
| | 90-107 | 4 | 4 | 0.00085197 | High | 2102.94226 | [K].IGCGENLFMSSQPYAWSR.[V] | | |
| | 215-224 | 2 | 1 | 0.00923644 | High | 1615.75278 | [K].QNACQTEWMK.[S] | | |
| ENSNNAP00000012975.1 | 64-83 | 1 | 1 | 0.00085197 | High | 2630.18627 | [R].GCGCPSVRNGIEINCCTTDR.[C] | Cobrotoxin | *P. mucrosquamatus* |
| P82849 | 1-26 | 1 | 6 | 0.00085197 | High | 3566.619 | [-].LECHNQQSSQTPTTTGCSGGENNCYK.[K] | Cobrotoxin II | *N. kaouthia* |
| P59275 | 1-25 | 1 | 2 | 0.00085197 | High | 3871.88293 | [-].LECHNQQSSQTPTTKTCSGETNCYK.[K] | Cobrotoxin-b | *N. kaouthia* |

| | | | | | | | | | |
|---|---|---|---|---|---|---|---|---|---|
| | 1-25 | 1 | 2 | 0.00085197 | High | 3567.67578 | [-].LECHNQQSSQTPTTKTCSGETNCYK.[K] | | |
| | 1-15 | 1 | 13 | 0.00085197 | High | 2367.22183 | [-].LECHNQQSSQTPTTK.[T] | | |
| | 1-15 | 1 | 2 | 0.00085197 | High | 2063.01468 | [-].LECHNQQSSQTPTTK.[T] | | |
| Q53B46 | 60-67 | 4 | 14 | 0.00085197 | High | 1570.85769 | [R].GCIDICPK.[S] | Beta-cardiotoxin CTX15 | *O. hannah* |
| P60305 | 24-36 | 10 | 38 | 0.00085197 | High | 2209.20386 | [K].MFMMSDLTIPVKR.[G] | Cytotoxin 1 | *N. kaouthia* |
| | 24-36 | 10 | 84 | 0.00085197 | High | 2193.20894 | [K].MFMMSDLTIPVKR.[G] | | |
| | 24-36 | 10 | 211 | 0.00085197 | High | 2177.21403 | [K].MFMMSDLTIPVKR.[G] | | |
| | 24-36 | 10 | 14 | 0.00085197 | High | 1904.99671 | [K].MFMMSDLTIPVKR.[G] | | |
| | 24-36 | 10 | 32 | 0.00085197 | High | 1889.0018 | [K].MFMMSDLTIPVKR.[G] | | |
| | 24-36 | 10 | 12 | 0.00085197 | High | 1873.00688 | [K].MFMMSDLTIPVKR.[G] | | |
| | 24-36 | 10 | 2 | 0.00085197 | High | 1584.79465 | [K].MFMMSDLTIPVKR.[G] | | |
| | 24-36 | 10 | 3 | 0.00085197 | High | 1568.79974 | [K].MFMMSDLTIPVKR.[G] | | |
| | 24-35 | 10 | 35 | 0.00085197 | High | 2053.10275 | [K].MFMMSDLTIPVK.[R] | | |
| | 24-35 | 10 | 107 | 0.00085197 | High | 2037.10783 | [K].MFMMSDLTIPVK.[R] | | |
| | 24-35 | 10 | 75 | 0.00085197 | High | 2021.11292 | [K].MFMMSDLTIPVK.[R] | | |
| | 24-35 | 10 | 2 | 0.00085197 | High | 1764.89052 | [K].MFMMSDLTIPVK.[R] | | |
| | 24-35 | 10 | 21 | 0.00085197 | High | 1748.8956 | [K].MFMMSDLTIPVK.[R] | | |
| | 24-35 | 10 | 13 | 0.00085197 | High | 1716.90577 | [K].MFMMSDLTIPVK.[R] | | |
| | 24-35 | 10 | 2 | 0.00085197 | High | 1428.69354 | [K].MFMMSDLTIPVK.[R] | | |
| | 24-35 | 10 | 3 | 0.00085197 | High | 1412.69863 | [K].MFMMSDLTIPVK.[R] | | |
| | 24-35 | 10 | 37 | 0.00085197 | High | 1732.90069 | [K].MFMMSDLTIPVK.[R] | | |
| | 3-12 | 8 | 2 | 0.00085197 | High | 1752.06973 | [K].CNKLIPIASK.[T] | | |
| | 3-12 | 8 | 1 | 0.00085197 | High | 2056.27688 | [K].CNKLIPIASK.[T] | | |
| | 1-12 | 1 | 1 | 0.00085197 | High | 2601.66305 | [-].LKCNKLIPIASK.[T] | | |
| | 6-18 | 6 | 4 | 0.00085197 | High | 2268.39297 | [K].LIPIASKTCPAGK.[N] | | |

| | | | | | | | | | |
|---|---|---|---|---|---|---|---|---|---|
| | 6-18 | 6 | 1 | 0.00085197 | High | 1964.18582 | [K].LIPIASKTCPAGK.[N] | | |
| | 6-12 | 8 | 137 | 0.00085197 | High | 1349.90119 | [K].LIPIASK.[T] | | |
| | 6-12 | 8 | 7 | 0.0058016 | High | 1045.69405 | [K].LIPIASK.[T] | | |
| P86541 | 19-35 | 1 | 1 | 0.00454195 | High | 2955.66901 | [K].DLCYKMYMVATPKVPVK.[R] | Cytotoxin 10 | *N. naja* |
| Q9W6W6 | 46-53 | 1 | 2 | 0.00085197 | High | 1484.91546 | [K].IFMVAAPK.[V] | Cytotoxin 10 | *N. atra* |
| | 46-53 | 1 | 3 | 0.00085197 | High | 1500.91038 | [K].IFMVAAPK.[V] | | |
| ENSNNAP00000013731.1 | 45-56 | 4 | 4 | 0.00165734 | High | 1989.14085 | [K].MFMVSDLTIPVK.[R] | cytotoxin 11 | *N. naja* |
| Q98956 | 72-81 | 1 | 17 | 0.00085197 | High | 1622.69321 | [K].YVCCNTDICN.[-] | Cytotoxin 1b | *N. atra* |
| | 24-39 | 1 | 1 | 0.00085197 | High | 2656.54586 | [K].CNKLVPIASKTCPAGK.[N] | | |
| P85429 | 6-15 | 1 | 1 | 0.0058016 | High | 1541.87209 | [K].LVPLFYKTCP.[-] | Cytotoxin 1f | *N. atra* |
| P01442 | 45-65 | 4 | 1 | 0.00085197 | High | 3363.85934 | [K].MFMVSNLTVPVKRGCIDVCPK.[NS] | Cytotoxin 2 | *N. atra* |
| | 45-57 | 4 | 114 | 0.00085197 | High | 2146.23721 | [K].MFMVSNLTVPVKR.[G] | | |
| | 45-57 | 4 | 125 | 0.00085197 | High | 2130.24229 | [K].MFMVSNLTVPVKR.[G] | | |
| | 45-57 | 4 | 26 | 0.00085197 | High | 1858.02498 | [K].MFMVSNLTVPVKR.[G] | | |
| | 45-57 | 4 | 67 | 0.00085197 | High | 1842.03006 | [K].MFMVSNLTVPVKR.[G] | | |
| | 45-57 | 4 | 25 | 0.00085197 | High | 1826.03515 | [K].MFMVSNLTVPVKR.[G] | | |
| | 45-57 | 4 | 4 | 0.00085197 | High | 1537.82292 | [K].MFMVSNLTVPVKR.[G] | | |
| | 45-56 | 4 | 202 | 0.00085197 | High | 1990.1361 | [K].MFMVSNLTVPVK.[R] | | |
| | 45-56 | 4 | 197 | 0.00085197 | High | 1974.14118 | [K].MFMVSNLTVPVK.[R] | | |
| | 45-56 | 4 | 35 | 0.00085197 | High | 1701.92387 | [K].MFMVSNLTVPVK.[R] | | |
| | 45-56 | 4 | 96 | 0.00085197 | High | 1685.92895 | [K].MFMVSNLTVPVK.[R] | | |
| | 45-56 | 4 | 54 | 0.00085197 | High | 1669.93404 | [K].MFMVSNLTVPVK.[R] | | |
| | 45-56 | 4 | 2 | 0.00085197 | High | 1381.72181 | [K].MFMVSNLTVPVK.[R] | | |
| | 45-56 | 4 | 1 | 0.00085197 | High | 1365.72689 | [K].MFMVSNLTVPVK.[R] | | |
| | 45-57 | 4 | 3 | 0.00085197 | High | 1521.828 | [K].MFMVSNLTVPVKR.[G] | | |

| | | | | | | | | | |
|---|---|---|---|---|---|---|---|---|---|
| | 40-56 | 4 | 1 | 0.0058016 | High | 2652.45711 | [K].NLCYKMFMVSNL TVPVK.[R] | | |
| Q9DG H9 | 45-52 | 2 | 3 | 0.0008519 7 | High | 1514.92603 | [K].IFMVATPK.[V] | Cytotoxin 2 | *N. kaouthia* |
| P01445 | 24-36 | 7 | 1 | 0.0008519 7 | High | 2145.25319 | [K].MFMVSNKTVPVK R.[G] | Cytotoxin 2 | *N. kaouthia* |
| | 24-35 | 7 | 15 | 0.0008519 7 | High | 1989.15208 | [K].MFMVSNKTVPVK. [R] | | |
| | 24-35 | 7 | 1 | 0.0058016 | High | 1684.94493 | [K].MFMVSNKTVPVK. [R] | | |
| | 24-35 | 7 | 3 | 0.0071623 9 | High | 1700.93985 | [K].MFMVSNKTVPVK. [R] | | |
| P01463 | 24-35 | 2 | 1 | 0.0032467 | High | 2005.118 | [K].MYMVATPMIPVK. [R] | Cytotoxin 2 | *N. nivea* |
| | 24-36 | 2 | 1 | 0.0045419 5 | High | 2145.2242 | [K].MYMVATPMIPVK R.[G] | | |
| O93472 | 58-65 | 1 | 1 | 0.0045419 5 | High | 1559.87472 | [R].GCIDVYPK.[S] | Cytotoxin 2c | *N. kaouthia* |
| P01452 | 51-60 | 1 | 1 | 0.0071623 9 | High | 1638.69936 | [K].YVCCSTDRCN.[-] | Cytotoxin 4 | *N. mossambi ca* |
| O93473 | 45-56 | 1 | 1 | 0.0008519 7 | High | 2002.15472 | [K].MFMVAMPKVPV K.[R] | Cytotoxin 4a | *N. sputatrix* |
| | 40-56 | 1 | 1 | 0.0016573 4 | High | 2696.46557 | [K].NLCYKMFMVAM PKVPVK.[R] | | |
| | 45-52 | 1 | 1 | 0.0045419 5 | High | 1562.87525 | [K].MFMVAMPK.[V] | | |
| | 45-56 | 1 | 7 | 0.0045419 5 | High | 2018.14964 | [K].MFMVAMPKVPV K.[R] | | |
| | 45-56 | 1 | 1 | 0.0058016 | High | 1729.93741 | [K].MFMVAMPKVPV K.[R] | | |
| O73856 | 45-56 | 1 | 3 | 0.0045419 5 | High | 2018.14964 | [K].MYMVAMPKVPV K.[R] | Cytotoxin 4b | *N. sputatrix* |
| | 40-56 | 1 | 1 | 0.0058016 | High | 3000.67271 | [K].NLCYKMYMVAM PKVPVK.[R] | | |
| | 45-56 | 1 | 4 | 0.0064723 9 | High | 2034.14455 | [K].MYMVAMPKVPV K.[R] | | |
| P07525 | 24-35 | 2 | 1 | 0.0008519 7 | High | 1714.93774 | [K].MFMVSNKMVPVK .[R] | Cytotoxin 5 | *N. atra* |
| | 24-35 | 2 | 1 | 0.0008519 7 | High | 2323.35203 | [K].MFMVSNKMVPVK .[R] | | |
| | 31-44 | 2 | 2 | 0.0016573 4 | High | 2571.47533 | [K].MVPVKRGCIDVC PK.[SN] | | |
| P01457 | 19-35 | 1 | 1 | 0.0058016 | High | 2615.38909 | [K].NLCYKMYMVSSS TVPVK.[R] | Cytotoxin 5 | *N. haje haje* |
| P24779 | 37-50 | 2 | 2 | 0.0008519 7 | High | 1879.01005 | [R].GCIDACPKNSLLV K.[Y] | Cytotoxin 5 | *N. kaouthia* |
| | 37-50 | 2 | 3 | 0.0016573 4 | High | 2183.2172 | [R].GCIDACPKNSLLV K.[Y] | | |

| | | | | | | | | | |
|---|---|---|---|---|---|---|---|---|---|
| | 37-44 | 2 | 1 | 0.00246457 | High | 1528.81074 | [R].GCIDACPK.[N] | | |
| P25517 | 37-50 | 1 | 2 | 0.00085197 | High | 2218.22195 | [R].GCIDVCPKSSFLVK.[Y] | Cytotoxin 5 | *N. mossambica* |
| Q98965 | 40-56 | 1 | 1 | 0.00085197 | High | 2727.47924 | [K].NLCYKMFMVAAQRFPVK.[R] | Cytotoxin 6 | *N. atra* |
| | 24-33 | 4 | 16 | 0.00085197 | High | 1888.06465 | [K].CNQLIPPFYK.[TA] | | |
| | 24-33 | 4 | 25 | 0.00085197 | High | 1583.8575 | [K].CNQLIPPFYK.[TA] | | |
| P01465 | 37-50 | 4 | 1 | 0.00085197 | High | 2170.18557 | [R].GCIDVCPKDSALVK.[Y] | Cytotoxin 6 | *N. annulifera* |
| | 37-50 | 4 | 3 | 0.00085197 | High | 2474.39271 | [R].GCIDVCPKDSALVK.[Y] | | |
| | 24-35 | 3 | 1 | 0.00923644 | High | 1684.91594 | [K].MYMVATPMLPVK.[R] | | |
| P49122 | 59-66 | 1 | 3 | 0.00085197 | High | 1196.58353 | [R].GCAATCPR.[S] | Cytotoxin 7 | *N. atra* |
| | 46-53 | 1 | 3 | 0.00085197 | High | 1830.2033 | [K].ATLKFPLK.[F] | | |
| | 46-57 | 1 | 3 | 0.00085197 | High | 2605.695 | [K].ATLKFPLKFPVK.[R] | | |
| | 24-40 | 1 | 34 | 0.00085197 | High | 2687.35655 | [K].CHNTQLPFIYNTCPEGK.[N] | | |
| | 24-40 | 1 | 7 | 0.00085197 | High | 2383.1494 | [K].CHNTQLPFIYNTCPEGK.[N] | | |
| | 58-66 | 1 | 1 | 0.0058016 | High | 1352.68464 | [K].RGCAATCPR.[S] | | |
| O73859 | 26-38 | 2 | 10 | 0.00085197 | High | 2177.22526 | [K].MFMMSNKTVPVKR.[G] | Cytotoxin 7 | *N. sputatrix* |
| | 26-37 | 2 | 6 | 0.00402361 | High | 2325.3313 | [K].MFMMSNKTVPVK.[R] | | |
| | 26-38 | 2 | 4 | 0.00857966 | High | 2481.43241 | [K].MFMMSNKTVPVKR.[G] | | |
| P86540 | 24-35 | 1 | 58 | 0.00085197 | High | 2006.13101 | [K].MYMVSDKTVPVK.[R] | Cytotoxin 8 | *N. naja* |
| | 24-30 | 1 | 6 | 0.00454195 | High | 1481.79878 | [K].MYMVSDK.[T] | | |
| | 24-35 | 1 | 3 | 0.00524997 | High | 2022.12592 | [K].MYMVSDKTVPVK.[R] | | |
| P60311 | 24-35 | 1 | 10 | 0.00085197 | High | 1670.91805 | [K].MFMVSDLTVPVK.[R] | Cytotoxin KJC3 | *N. sputatrix* |
| | 24-35 | 1 | 1 | 0.00085197 | High | 1366.71091 | [K].MFMVSDLTVPVK.[R] | | |
| | 24-35 | 1 | 16 | 0.00085197 | High | 1686.91297 | [K].MFMVSDLTVPVK.[R] | | |
| | 24-35 | 1 | 5 | 0.00085197 | High | 1702.90788 | [K].MFMVSDLTVPVK.[R] | | |

| | | | | | | | | | |
|---|---|---|---|---|---|---|---|---|---|
| | 24-35 | 1 | 19 | 0.00085197 | High | 1975.1252 | [K].MFMVSDLTVPVK.[R] | | |
| | 24-35 | 1 | 39 | 0.00085197 | High | 1991.12011 | [K].MFMVSDLTVPVK.[R] | | |
| | 24-36 | 1 | 23 | 0.00085197 | High | 2147.22122 | [K].MFMVSDLTVPVKR.[G] | | |
| | 24-36 | 1 | 59 | 0.00085197 | High | 2131.22631 | [K].MFMVSDLTVPVKR.[G] | | |
| | 24-36 | 1 | 1 | 0.00085197 | High | 1859.00899 | [K].MFMVSDLTVPVKR.[G] | | |
| | 24-36 | 1 | 10 | 0.00085197 | High | 1843.01408 | [K].MFMVSDLTVPVKR.[G] | | |
| | 24-36 | 1 | 6 | 0.00085197 | High | 1827.01916 | [K].MFMVSDLTVPVKR.[G] | | |
| | 24-36 | 1 | 1 | 0.00246457 | High | 1522.81202 | [K].MFMVSDLTVPVKR.[G] | | |
| P60308 | 19-31 | 9 | 4 | 0.00085197 | High | 2515.40552 | [K].NLCYKMFMVATPK.[V] | Cytotoxin SP15c | *N. atra* |
| | 24-35 | 9 | 1 | 0.00085197 | High | 2260.37415 | [K].MFMVATPKVPVK.[R] | | |
| | 24-35 | 9 | 2 | 0.00085197 | High | 1972.16192 | [K].MFMVATPKVPVK.[R] | | |
| | 24-35 | 9 | 1 | 0.00085197 | High | 1956.167 | [K].MFMVATPKVPVK.[R] | | |
| | 24-31 | 9 | 113 | 0.00085197 | High | 1548.87736 | [K].MFMVATPK.[V] | | |
| | 24-31 | 9 | 665 | 0.00085197 | High | 1532.88245 | [K].MFMVATPK.[V] | | |
| | 24-31 | 9 | 21 | 0.00085197 | High | 1260.66513 | [K].MFMVATPK.[V] | | |
| | 24-31 | 9 | 85 | 0.00085197 | High | 1244.67022 | [K].MFMVATPK.[V] | | |
| | 24-31 | 9 | 41 | 0.00085197 | High | 1228.6753 | [K].MFMVATPK.[V] | | |
| | 37-44 | 1 | 1 | 0.00085197 | High | 1628.86317 | [R].ECIDVCPK.[S] | | |
| | 37-44 | 1 | 9 | 0.0032467 | High | 1324.65602 | [R].ECIDVCPK.[S] | | |
| | 24-36 | 9 | 1 | 0.00402361 | High | 2416.47526 | [K].MFMVATPKVPVKR.[EG] | | |
| | 36-44 | 1 | 1 | 0.00454195 | High | 1784.96428 | [K].RECIDVCPK.[S] | | |
| | 24-31 | 9 | 2 | 0.00454195 | High | 924.46816 | [K].MFMVATPK.[V] | | |
| P62377 | 53-62 | 8 | 8 | 0.00085197 | High | 1914.90036 | [K].YVCCSTDKCN.[-] | Cytotoxin-like basic protein | *N. naja* |
| | 53-62 | 8 | 6 | 0.00085197 | High | 1610.69321 | [K].YVCCSTDKCN.[-] | | |
| | 53-60 | 8 | 9 | 0.00085197 | High | 1640.82678 | [K].YVCCSTDK.[C] | | |

| | | | | | | | | | |
|---|---|---|---|---|---|---|---|---|---|
| | 14-24 | 9 | 1 | 0.00085197 | High | 2266.2504 | [K].TCPEGKNLCFK.[AMT] | | |
| | 14-24 | 9 | 1 | 0.00085197 | High | 1962.04326 | [K].TCPEGKNLCFK.[AMT] | | |
| | 39-52 | 8 | 6 | 0.00085197 | High | 2460.35191 | [R].GCADNCPKNSALLK.[Y] | | |
| | 39-46 | 8 | 5 | 0.00085197 | High | 1529.7696 | [R].GCADNCPK.[N] | | |
| | 3-13 | 8 | 28 | 0.00085197 | High | 2029.11847 | [K].CHNTQLPFIYK.[T] | | |
| | 1-13 | 8 | 12 | 0.00085197 | High | 1724.91133 | [K].CHNTQLPFIYK.[T] | | |
| | 1-13 | 3 | 1 | 0.00085197 | High | 2574.50464 | [-].LKCHNTQLPFIYK.[T] | | |
| | 29-37 | 6 | 4 | 0.00524997 | High | 2016.319 | [KR].KFPLKFPVK.[R] | | |
| | 53-60 | 8 | 7 | 0.0058016 | High | 1336.61964 | [K].YVCCSTDK.[C] | | |
| | 38-46 | 8 | 1 | 0.0058016 | High | 1685.87071 | [K].RGCADNCPK.[N] | | |
| P25669 | 50-69 | 5 | 1 | 0.00857966 | High | 2676.2023 | [RK].TGVDIQCCSTDDCDPFPTRK.[R] | Long neurotoxin 2 | *N. naja* |
| Q2VBP3 | 59-72 | 1 | 2 | 0.00165734 | High | 2157.19032 | [R].VIELGCTATCPTVK.[H] | Long neurotoxin LNTX37 | *O. hannah* |
| P18328 | 62-83 | 1 | 1 | 0.00085197 | High | 3117.48296 | [K].GCAATCPKVDNNDPIRCCGTDK.[C] | Muscarinic toxin 2 | *N. angusticeps* |
| P82462 | 49-62 | 2 | 1 | 0.00085197 | High | 2364.22956 | [K].LQNRDVIFCCSTDK.[C] | Muscarinic toxin-like protein 1 | *N. kaouthia* |
| | 53-62 | 2 | 58 | 0.00085197 | High | 1852.94288 | [R].DVIFCCSTDK.[C] | | |
| | 53-62 | 2 | 4 | 0.00085197 | High | 1548.73573 | [R].DVIFCCSTDK.[C] | | |
| | 53-65 | 2 | 35 | 0.00085197 | High | 2240.10052 | [R].DVIFCCSTDKCNL.[-] | | |
| | 8-35 | 1 | 4 | 0.00085197 | High | 3577.7051 | [K].FLFSETTETCPDGQNVCFNQAHLIYPGK.[Y] | | |
| P82463 | 53-65 | 1 | 1 | 0.00085197 | High | 2222.07469 | [R].DVIECCSTDKCNL.[-] | Muscarinic toxin-like protein 2 | *N. kaouthia* |
| P82464 | 39-59 | 1 | 1 | 0.00085197 | High | 2666.20946 | [K].RGCTFTCPELRPTGIYVYCCR.[R] | Muscarinic toxin-like protein 3 | *N. kaouthia* |
| | 39-59 | 1 | 1 | 0.00085197 | High | 2970.41661 | [K].RGCTFTCPELRPTGIYVYCCR.[R] | | |
| | 40-59 | 2 | 7 | 0.00085197 | High | 2814.3155 | [R].GCTFTCPELRPTGIYVYCCR.[R] | | |
| | 40-59 | 2 | 1 | 0.00085197 | High | 2510.10835 | [R].GCTFTCPELRPTGIYVYCCR.[R] | | |
| Q9PSN6 | 1-27 | 1 | 8 | 0.00085197 | High | 3986.90987 | [-].LECHDQQSSQTPTTTGCSGGETNCYKK.[R] | Neurotoxin 3 | *N. sputatrix* |

| | | | | | | | | | |
|---|---|---|---|---|---|---|---|---|---|
| | 1-26 | 1 | 48 | 0.00085197 | High | 3554.60776 | [-].LECHDQQSSQTPTTTGCSGGETNCYK.[K] | | |
| | 1-26 | 1 | 2 | 0.00085197 | High | 3292.41118 | [-].LECHDQQSSQTPTTTGCSGGETNCYK.[K] | | |
| | 1-26 | 1 | 3 | 0.00085197 | High | 3250.40062 | [-].LECHDQQSSQTPTTTGCSGGETNCYK.[K] | | |
| Q9DEQ3 | 60-74 | 1 | 4 | 0.00085197 | High | 2084.07003 | [K].RGCTFTCPELRPTGK.[Y] | Neurotoxin homolog NL1 | *N. atra* |
| | 60-74 | 1 | 4 | 0.00085197 | High | 2388.27717 | [K].RGCTFTCPELRPTGK.[Y] | | |
| | 31-47 | 1 | 16 | 0.00085197 | High | 2805.33956 | [R].TPETTEICPDSWYFCYK.[I] | | |
| | 31-47 | 1 | 6 | 0.00085197 | High | 2501.13241 | [R].TPETTEICPDSWYFCYK.[I] | | |
| | 61-74 | 1 | 7 | 0.00085197 | High | 1927.96892 | [R].GCTFTCPELRPTGK.[Y] | | |
| | 61-74 | 1 | 30 | 0.00085197 | High | 2232.17606 | [R].GCTFTCPELRPTGK.[Y] | | |
| Q9W717 | 74-81 | 4 | 9 | 0.00085197 | High | 1346.65161 | [RK].VYVDCCAR.[D] | Neurotoxin-like protein NTL2 | *N. atra* |
| P85520 | 53-62 | 7 | 35 | 0.00085197 | High | 1848.9327 | [R].EIVECCSTDK.[C-] | Oxiana weak toxin | *N. oxiana* |
| | 53-65 | 6 | 6 | 0.00085197 | High | 2260.06519 | [R].EIVECCSTDKCNH.[-] | | |
| | 10-22 | 1 | 1 | 0.0032467 | High | 2274.17271 | [K].YCNKVHTCRNGEK.[I] | | |
| Q9YGI4 | 62-83 | 1 | 1 | 0.00085197 | High | 3209.47616 | [R].GCAATCPETKPRDMVECCSTDR.[C] | Probable weak neurotoxin NNAM2 | *N. atra* |
| | 62-73 | 1 | 29 | 0.00085197 | High | 1956.02867 | [R].GCAATCPETKPR.[D] | | |
| | 62-73 | 1 | 3 | 0.00085197 | High | 1651.82153 | [R].GCAATCPETKPR.[D] | | |
| | 53-73 | 1 | 2 | 0.00165734 | High | 2840.50952 | [R].RPFSLRYIRGCAATCPETKPR.[D] | | |
| P60774 | 1-15 | 3 | 1 | 0.00085197 | High | 2033.00412 | [-].LECHNQQSSQAPTTK.[T] | Short neurotoxin 1 | *N. samarensis* |
| | 1-15 | 3 | 14 | 0.00085197 | High | 2337.21126 | [-].LECHNQQSSQAPTTK.[T] | | |
| | 1-25 | 3 | 2 | 0.00085197 | High | 3841.87236 | [-].LECHNQQSSQAPTTKTCSGETNCYK.[K] | | |
| | 1-25 | 3 | 1 | 0.00085197 | High | 3537.66522 | [-].LECHNQQSSQAPTTKTCSGETNCYK.[K] | | |
| P01427 | 1-25 | 1 | 1 | 0.00454195 | High | 3867.88801 | [-].LECHNQQSSQPPTTKTCSGETNCYK.[K] | Short neurotoxin 1 | *N. oxiana* |
| P14613 | 1-27 | 1 | 1 | 0.00085197 | High | 3707.77075 | [-].LECHNQQSIQTPTTTGCSGGETNCYKK.[R] | Short neurotoxin 1 | *N. kaouthia* |
| | 1-26 | 1 | 1 | 0.00085197 | High | 3579.67578 | [-].LECHNQQSIQTPTTTGCSGGETNCYK.[K] | | |

| | | | | | | | | | |
|---|---|---|---|---|---|---|---|---|---|
| P01431 | 17-26 | 1 | 3 | 0.00085197 | High | 1783.85987 | [R].CSGGETNCYK.[K] | Short neurotoxin 1 | *N. mossambica* |
| | 17-26 | 1 | 3 | 0.00085197 | High | 1479.65273 | [R].CSGGETNCYK.[K] | | |
| | 49-59 | 5 | 9 | 0.00085197 | High | 1642.78481 | [K].GIELNCCTTDR.[C] | | |
| | 49-59 | 5 | 4 | 0.00085197 | High | 1338.57766 | [K].GIEINCCTTDR.[C] | | |
| P68418 | 1-26 | 2 | 1 | 0.00085197 | High | 3877.90875 | [-].LECHNQQSSQPPTTKTCPGETNCYK.[K] | Short neurotoxin 1 | *N. haje haje* |
| | 1-26 | 2 | 1 | 0.0058016 | High | 3701.79657 | [-].LECHNQQSSQPPTTKTCPGETNCYKK.[VR] | | |
| | 1-25 | 2 | 2 | 0.0058016 | High | 3311.50503 | [-].LECHNQQSSQPPTTKTCPGETNCYK.[K] | | |
| P01424 | 1-25 | 1 | 1 | 0.00923644 | High | 3607.65294 | [-].MECHNQQSSQPPTTKTCPGETNCYK.[K] | Short neurotoxin 1 | *N. melanoleuca* |
| P01432 | 1-26 | 1 | 1 | 0.00085197 | High | 3602.63164 | [-].LNCHNQMSAQPPTTTRCSRWETNCYK.[K] | Short neurotoxin 3 | *N. mossambica* |
| | 1-26 | 1 | 6 | 0.00454195 | High | 3848.8333 | [-].LNCHNQMSAQPPTTTRCSRWETNCYK.[K] | | |
| E2IU01 | 24-37 | 3 | 27 | 0.00085197 | High | 2156.18115 | [KR].RVDLGCAATCPTVK.[T] | Three-finger toxin | *N. atra* |
| | 24-37 | 3 | 5 | 0.00085197 | High | 1851.974 | [KR].RVDLGCAATCPTVK.[T] | | |
| | 25-37 | 5 | 20 | 0.00085197 | High | 2000.08004 | [R].VDLGCAATCPTVK.[T] | | |
| | 25-37 | 5 | 14 | 0.00085197 | High | 1695.87289 | [R].VDLGCAATCPTVK.[T] | | |
| | 38-57 | 1 | 58 | 0.00085197 | High | 2643.19207 | [K].TGVDIQCCSTDNCNPFPTRP.[-] | | |
| | 14-21 | 1 | 2 | 0.00246457 | High | 985.36146 | [R].CDGFCGNR.[G] | | |
| E2ITZ9 | 47-60 | 2 | 35 | 0.00085197 | High | 2242.19278 | [K].VKPGVNLNCCTTDR.[C] | Three-finger toxin | *N. atra* |
| | 47-60 | 2 | 5 | 0.00085197 | High | 1937.98563 | [K].VKPGVNLNCCTTDR.[C] | | |
| | 1-27 | 1 | 7 | 0.0032467 | High | 3873.79736 | [-].YTLECHNQQSSQTPTTKTCSGETNCYK.[K] | | |
| | 1-27 | 1 | 2 | 0.0032467 | High | 3831.78679 | [-].YTLECHNQQSSQTPTTKTCSGETNCYK.[K] | | |
| | 1-27 | 1 | 3 | 0.00524997 | High | 3569.59021 | [-].YTLECHNQQSSQTPTTKTCSGETNCYK.[K] | | |

| | | | | | | | | | |
|---|---|---|---|---|---|---|---|---|---|
| P85092 | 8-26 | 1 | 2 | 0.00246457 | High | 2810.39847 | [K].SIFGITTEDCPDGQNLCFK.[R] | Toxin AdTx1 | *N. angusticeps* |
| P29180 | 14-22 | 7 | 4 | 0.00085197 | High | 1665.92379 | [K].VHTCLNGEK.[I] | Weak neurotoxin 6 | *N. naja* |
| | 14-22 | 7 | 3 | 0.00085197 | High | 1361.71665 | [K].VHTCLNGEK.[I] | | |
| | 53-65 | 1 | 1 | 0.00085197 | High | 2259.08118 | [R].EIVQCCSTDKCNH.[-] | | |
| | 53-62 | 3 | 12 | 0.00085197 | High | 1847.94869 | [R].EIVQCCSTDK.[C] | | |
| | 41-49 | 5 | 14 | 0.00085197 | High | 1339.64177 | [R].GCADTCPVR.[K] | | |
| O42256 | 62-73 | 5 | 1 | 0.00085197 | High | 1579.8004 | [R].GCAATCPGTKPR.[D] | Weak neurotoxin 6 | *N. sputatrix* |
| | 62-73 | 5 | 6 | 0.00085197 | High | 1884.00754 | [R].GCAATCPGTKPR.[D] | | |
| | 62-86 | 4 | 5 | 0.00246457 | High | 3583.62464 | [R].GCAATCPGTKPRDMVECCSTDRCNR.[-] | | |
| Q2VBN2 | 62-71 | 4 | 4 | 0.00085197 | High | 1672.86423 | [R].GCAATCPEAK.[TS] | Weak neurotoxin WNTX34 | *O. hannah* |
| | 61-71 | 1 | 1 | 0.00524997 | High | 1828.96534 | [R].RGCAATCPEAK.[S] | | |
| P01401 | 19-26 | 1 | 4 | 0.00085197 | High | 1604.83465 | [R].NGENQCFK.[R] | Weak toxin CM-11 | *N. haje haje* |
| | 53-62 | 1 | 2 | 0.00454195 | High | 1586.74736 | [R].EIVECCTTDR.[C] | | |
| P25679 | 1-13 | 2 | 3 | 0.00085197 | High | 1629.6892 | [-].LTCLNCPEMFCGK.[F] | Weak toxin CM-9a | *N. kaouthia* |
| | 1-13 | 2 | 27 | 0.00085197 | High | 1933.89635 | [-].LTCLNCPEMFCGK.[F] | | |
| | 1-13 | 2 | 10 | 0.00085197 | High | 1949.89126 | [-].LTCLNCPEMFCGK.[F] | | |
| | 1-13 | 2 | 3 | 0.00085197 | High | 1975.90691 | [-].LTCLNCPEMFCGK.[F] | | |
| | 1-13 | 2 | 62 | 0.00085197 | High | 2238.10349 | [-].LTCLNCPEMFCGK.[F] | | |
| | 1-13 | 2 | 54 | 0.00085197 | High | 2254.09841 | [-].LTCLNCPEMFCGK.[F] | | |
| | 52-61 | 1 | 1 | 0.00085197 | High | 1880.90478 | [K].EMIECCSTDK.[C] | | |
| | 1-13 | 2 | 1 | 0.00454195 | High | 1645.68412 | [-].LTCLNCPEMFCGK.[F] | | |
| D9IX97 | 91-98 | 4 | 18 | 0.00085197 | High | 1478.79172 | [K].GSSCFGQK.[I] | Natriuretic peptide Na-NP | *N. atra* |
| | 91-98 | 4 | 2 | 0.00085197 | High | 1174.58457 | [K].GSSCFGQK.[I] | | |
| | 102-111 | 1 | 9 | 0.00085197 | High | 1359.65023 | [R].IGSMSGMGCR.[T] | | |

| | | | | | | | | | |
|---|---|---|---|---|---|---|---|---|---|
| | 89-98 | 1 | 1 | 0.00085197 | High | 1663.90814 | [K].GKGSSCFGQK.[I] | | |
| | 89-98 | 1 | 3 | 0.00085197 | High | 1968.11529 | [K].GKGSSCFGQK.[I] | | |
| | 102-111 | 1 | 1 | 0.00454195 | High | 1391.64006 | [R].IGSMSGMGCR.[T] | | |
| | 102-111 | 1 | 7 | 0.0058016 | High | 1375.64514 | [R].IGSMSGMGCR.[T] | | |
| V8NF35 | 138-148 | 1 | 1 | 0.00085197 | High | 1376.70892 | [R].SSNPADPASAR.[E] | Dipeptidyl peptidase 2 | *O. hannah* |
| V8P9G9 | 170-187 | 1 | 1 | 0.00085197 | High | 2825.5825 | [K].TTPNAKPVEITENGAENK.[I] | Venom dipeptidylpeptidase IV | *O. hannah* |
| | 363-371 | 1 | 1 | 0.00085197 | High | 1618.92959 | [R].IISDSQGYK.[H] | | |
| | 346-357 | 2 | 1 | 0.00085197 | High | 2024.06206 | [R].FQPSEPYFASDK.[I] | | |
| | 600-611 | 1 | 1 | 0.00085197 | High | 2358.31953 | [K].LFSEMSFVDKDK.[M] | | |
| ENSNNAP00000025792.1 | 33-41 | 1 | 1 | 0.00085197 | High | 1401.7922 | [R].RPPPAFQER.[F] | Dipeptidyl peptidase 7 | *P. textilis* |
| | 298-307 | 2 | 1 | 0.00085197 | High | 1715.96797 | [K].VGCELMLASK.[D] | | |
| | 236-245 | 2 | 1 | 0.00085197 | High | 1368.78063 | [K].DLSLIGAYGR.[I] | | |
| V8P395 | 104-111 | 1 | 5 | 0.00085197 | High | 1593.84381 | [K].VDCYDSVK.[G] | Glutathione peroxidase | *O. hannah* |
| | 102-111 | 1 | 7 | 0.00085197 | High | 2097.18303 | [R].AKVDCYDSVK.[G] | | |
| | 245-251 | 1 | 1 | 0.00454195 | High | 1356.83423 | [R].TNVSTVK.[N] | | |
| ENSNNAP00000008637.1 | 78-86 | 2 | 1 | 0.00085197 | High | 1634.98738 | [K].TFHIVPNAK.[E] | Hyaluronidase | *N. scutatus* |
| | 454-463 | 1 | 1 | 0.00085197 | High | 2239.23613 | [K].GLYCEEHYKK.[K] | | |
| | 454-462 | 1 | 1 | 0.00085197 | High | 1806.93403 | [K].GLYCEEHYK.[K] | | |
| | 443-453 | 1 | 2 | 0.00402361 | High | 1825.81433 | [K].NFMCQCYQGWK.[G] | | |
| ENSNNAP00000024830.1 | 95-109 | 1 | 1 | 0.00085197 | High | 2147.98431 | [R].GIVEECCFQSCDLAR.[L] | Insulin like growth factor 1 | *N. scutatus* |
| ENSNNAP00000006918.1 | 267-277 | 2 | 2 | 0.00085197 | High | 1802.99325 | [K].YGQPLPGYDGK.[G] | Insulin like growth factor binding protein 3 | *P. textilis* |

| | | | | | | | | | |
|---|---|---|---|---|---|---|---|---|---|
| | 87-100 | 1 | 3 | 0.00085197 | High | 2437.44823 | [R].TDEPKPLQALLDGK.[G] | | |
| | 87-100 | 1 | 1 | 0.00085197 | High | 2133.24109 | [R].TDEPKPLQALLDGK.[G] | | |
| | 53-76 | 1 | 3 | 0.00085197 | High | 3106.348 | [R].EPGCGCCLTCALQEGQPCGVYTER.[C] | | |
| | 215-224 | 2 | 1 | 0.00085197 | High | 1814.98137 | [R].EMEDTLNNLK.[I] | | |
| | 36-52 | 1 | 1 | 0.00085197 | High | 2654.39597 | [R].ALEQCKPMVSECPELVR.[E] | | |
| | 259-266 | 2 | 2 | 0.00085197 | High | 1695.84785 | [R].GYCWCVDK.[Y] | | |
| | 225-232 | 2 | 2 | 0.00085197 | High | 1215.77442 | [K].ILNVLSPR.[G] | | |
| V8N7H9 | 74-82 | 2 | 3 | 0.00085197 | High | 1539.75449 | [R].YHTIEECQR.[Y] | BPTI/Kunitz domain-containing protein-like | *O. hannah* |
| | 83-98 | 1 | 5 | 0.00085197 | High | 2510.29282 | [R].YCGHIEKPGFCPPSPR.[G] | | |
| | 58-71 | 1 | 2 | 0.00085197 | High | 1997.92812 | [K].SCEPFIYHGCPGNR.[N] | | |
| | 99-115 | 1 | 4 | 0.00085197 | High | 2150.94491 | [R].GASGPCQASCLHDGNCR.[E] | | |
| | 165-178 | 1 | 12 | 0.00085197 | High | 2129.07298 | [K].FTYGGCAGNANNFK.[R] | | |
| | 165-178 | 1 | 1 | 0.00524997 | High | 1824.86583 | [K].FTYGGCAGNANNFK.[R] | | |
| P20229 | 47-57 | 2 | 1 | 0.00085197 | High | 1628.76916 | [R].TIDECNRTCVG.[-] | Kunitz-type serine protease inhibitor | *N. naja* |
| | 33-44 | 1 | 6 | 0.00085197 | High | 1589.78138 | [K].FIYGGCGGNANR.[F] | | |
| ENSNNAP00000023266.1 | 91-102 | 1 | 1 | 0.00085197 | High | 1932.14837 | [K].APPDLITICPVK.[C] | Kunitz-type serine protease inhibitor 4-like | *N. scutatus* |
| A8QL51 | 175-183 | 2 | 1 | 0.00085197 | High | 1738.91434 | [K].NDDLFSYEK.[R] | L-amino acid oxidase | *B. multicinctus* |
| ENSNNAP00000002373.1 | 24-43 | 2 | 26 | 0.00085197 | High | 2821.33099 | [R].SPLEECFQQNDYEEFLEIAR.[N] | L-amino-acid oxidase | *N. atra* |
| A8QL58 | 91-98 | 1 | 5 | 0.00085197 | High | 1321.71836 | [R].VITYHNDR.[E] | L-amino-acid oxidase | *N. atra* |
| V8P5W4 | 146-158 | 2 | 1 | 0.00085197 | High | 1726.82435 | [K].NDFYATGESYAGK.[Y] | serine carboxypeptidase | *O. hannah* |
| | 370-382 | 2 | 1 | 0.00085197 | High | 2107.17916 | [R].IHDKDPEVAGYVR.[Q] | | |
| | 394-406 | 2 | 1 | 0.00085197 | High | 1742.9145 | [R].GGGHILPYDQPER.[A] | | |

| | | | | | | | | | |
|---|---|---|---|---|---|---|---|---|---|
| | 383-393 | 2 | 1 | 0.0058016 | High | 1641.92836 | [R].QVGEFYQVIVR.[G] | | |
| ENSNNAP00000011792.1 | 180-189 | 2 | 1 | 0.00085197 | High | 1774.11225 | [R].LVVINAVYFK.[G] | Serpin | *N. scutatus* |
| | 329-340 | 1 | 2 | 0.00085197 | High | 2030.17776 | [R].SENIYVSQLLQK.[A] | | |
| | 352-363 | 2 | 1 | 0.00085197 | High | 1436.83921 | [K].ASAATTAIVTAR.[S] | | |
| | 341-351 | 2 | 1 | 0.00085197 | High | 2089.23209 | [K].AKIEVSEDGTK.[A] | | |
| | 109-120 | 2 | 1 | 0.00085197 | High | 1855.12969 | [K].IANIVFANSGLK.[M] | | |
| | 196-202 | 2 | 1 | 0.00454195 | High | 1471.84005 | [R].FQPENTK.[K] | | |
| A0A2I4HXH5 | 302-316 | 1 | 1 | 0.00085197 | High | 2389.31533 | [R].MKVQLQNYSSQEIGR.[T] | Snake venom 5'-nucleotidase | *N. atra* |
| | 304-316 | 1 | 2 | 0.00085197 | High | 1825.97274 | [K].VQLQNYSSQEIGR.[T] | | |
| | 75-84 | 1 | 2 | 0.00085197 | High | 1551.82726 | [R].EVVHFMNSLR.[Y] | | |
| | 75-84 | 1 | 5 | 0.00085197 | High | 1535.83235 | [R].EVVHFMNSLR.[Y] | | |
| | 190-203 | 1 | 12 | 0.00085197 | High | 1909.95835 | [K].IIALGHSGFMEDCR.[I] | | |
| | 190-203 | 1 | 5 | 0.00085197 | High | 1925.95327 | [K].IIALGHSGFMEDCR.[I] | | |
| | 75-84 | 1 | 1 | 0.00454195 | High | 1231.6252 | [R].EVVHFMNSLR.[Y] | | |
| V8NYW9 | 141-148 | 1 | 1 | 0.00085197 | High | 1559.92886 | [K].VVYDLSQK.[A] | 5'-nucleotidase | *O. hannah* |
| A8QL53 | 254-267 | 4 | 25 | 0.00085197 | High | 2231.23898 | [R].FPCAQLLEPGVYTK.[V] | Snake venom serine protease NaSP | *N. atra* |
| | 254-267 | 4 | 2 | 0.00524997 | High | 1927.03184 | [R].FPCAQLLEPGVYTK.[V] | | |
| P86545 | 1-12 | 1 | 1 | 0.00085197 | High | 1788.84068 | [-].IGGFECNEHEHR.[S] | Thrombin-like enzyme | *N. naja* |
| V8NCP7 | 144-162 | 1 | 1 | 0.00085197 | High | 2755.48452 | [K].ESGATTNIFFKPPCVSVHR.[C] | Vascular endothelial growth factor C | *O. hannah* |
| | 136-143 | 2 | 1 | 0.00085197 | High | 1454.87101 | [R].EVTVDVGK.[E] | | |
| | 352-359 | 2 | 1 | 0.00085197 | High | 1542.92478 | [K].HQPLNPTK.[C] | | |
| | 325-334 | 2 | 2 | 0.00085197 | High | 1696.99115 | [K].LLPSSCGLNK.[E] | | |
| | 410-416 | 2 | 1 | 0.00165734 | High | 1561.86924 | [R].CVPTYWK.[L] | | |

| | | | | | | | | | |
|---|---|---|---|---|---|---|---|---|---|
| P61899 | 58-67 | 3 | 2 | 0.00085197 | High | 1431.69698 | [RK].NPNPEPSGCR.[G] | Venom nerve growth factor | *N. kaouthia* |
| | 24-45 | 3 | 4 | 0.00085197 | High | 3290.80821 | [K].TTATDIKGNTVTVMENVNLDNK.[V] | | |
| | 1-23 | 3 | 1 | 0.00085197 | High | 3211.60861 | [-R].EDHPVHNLGEHSVCDSVSAWVTK.[T] | | |
| | 87-98 | 4 | 26 | 0.00085197 | High | 1683.84437 | [K].ALTMEGNQASWR.[F] | | |
| | 87-98 | 4 | 22 | 0.00085197 | High | 1667.84945 | [K].ALTMEGNQASWR.[F] | | |
| | 56-67 | 2 | 5 | 0.00085197 | High | 2024.02973 | [K].CKNPNPEPSGCR.[G] | | |
| | 56-67 | 2 | 3 | 0.00085197 | High | 1719.82259 | [K].CKNPNPEPSGCR.[G] | | |
| | 102-112 | 1 | 9 | 0.00085197 | High | 1902.06841 | [R].IETACVCVITK.[K] | | |
| | 102-112 | 1 | 1 | 0.00085197 | High | 1597.86127 | [R].IETACVCVITK.[K] | | |
| | 31-45 | 3 | 5 | 0.00085197 | High | 2272.20987 | [K].GNTVTVMENVNLDNK.[V] | | |
| | 31-45 | 3 | 5 | 0.00085197 | High | 2256.21495 | [K].GNTVTVMENVNLDNK.[V] | | |
| | 68-86 | 3 | 1 | 0.00085197 | High | 2260.98154 | [R].GIDSSHWNSYCTETDTFIK.[A] | | |
| | 68-86 | 3 | 2 | 0.00085197 | High | 2869.39583 | [R].GIDSSHWNSYCTETDTFIK.[A] | | |
| Q5YF90 | 184-193 | 4 | 1 | 0.00085197 | High | 1401.7228 | [RK].NPNPVPSGCR.[G] | Venom nerve growth factor 1 | *N. sputatrix* |
| | 175-181 | 9 | 27 | 0.00085197 | High | 1570.8761 | [RK].QYFFETK.[C] | | |
| | 228-238 | 4 | 1 | 0.00085197 | High | 1597.83611 | [R].IDTACVCVISR.[K] | | |
| A0A2D0TC04 | 501-510 | 3 | 2 | 0.00085197 | High | 1438.76498 | [K].NPFYNPSPAK.[E] | Venom phosphodiesterase | *N. atra* |
| | 501-510 | 3 | 12 | 0.00085197 | High | 1742.97212 | [K].NPFYNPSPAK.[E] | | |
| | 554-573 | 3 | 3 | 0.00085197 | High | 2915.54416 | [K].MQSEADNLPYGRPHVLQHSK.[Y] | | |
| | 554-573 | 3 | 1 | 0.00085197 | High | 2627.33193 | [K].MQSEADNLPYGRPHVLQHSK.[Y] | | |
| | 203-222 | 3 | 2 | 0.00085197 | High | 2575.31654 | [R].NAAWWGGQPIWHTASYQGLK.[A] | | |
| | 270-287 | 3 | 4 | 0.00085197 | High | 2714.42811 | [K].RPDFSTLYIEEPDTTGHK.[F] | | |
| | 366-381 | 3 | 2 | 0.00085197 | High | 2481.3521 | [K].NVPKDFYTFDSEAIVK.[K] | | |
| | 546-553 | 3 | 12 | 0.00085197 | High | 1522.94485 | [R].LNLIDQAK.[M] | | |

| | | | | | | |
|---|---|---|---|---|---|---|
| 65-80 | 3 | 1 | 0.00085197 | High | 2524.25234 | [R].MANVLCSCSEDCLTKK.[D] |
| 65-79 | 3 | 8 | 0.00085197 | High | 2396.15738 | [R].MANVLCSCSEDCLTK.[K] |
| 65-79 | 3 | 1 | 0.00085197 | High | 2107.94515 | [R].MANVLCSCSEDCLTK.[K] |
| 65-79 | 3 | 2 | 0.00085197 | High | 2091.95023 | [R].MANVLCSCSEDCLTK.[K] |
| 65-80 | 3 | 1 | 0.00085197 | High | 2540.24726 | [R].MANVLCSCSEDCLTKK.[D] |
| 677-688 | 3 | 2 | 0.00085197 | High | 2127.22465 | [R].LWNYFHSTLLPK.[Y] |
| 65-80 | 3 | 6 | 0.00085197 | High | 2828.45949 | [R].MANVLCSCSEDCLTKK.[D] |
| 433-446 | 3 | 5 | 0.00085197 | High | 2243.06829 | [K].YCSGGTHGYDNEFK.[S] |
| 574-581 | 3 | 3 | 0.00085197 | High | 1670.95437 | [K].YCLLHQTK.[Y] |
| 447-460 | 3 | 3 | 0.00085197 | High | 2129.1709 | [K].SMEAIFLAHGPGFK.[E] |
| 776-785 | 3 | 4 | 0.00085197 | High | 1548.79774 | [K].SPDNLWVEER.[M] |
| 447-460 | 3 | 4 | 0.00085197 | High | 2113.17599 | [K].SMEAIFLAHGPGFK.[E] |
| 603-619 | 3 | 3 | 0.00085197 | High | 2394.33065 | [K].SLVKPTSAPPSASDCLR.[L] |
| 447-460 | 3 | 1 | 0.00085197 | High | 1808.96884 | [K].SMEAIFLAHGPGFK.[E] |
| 746-756 | 3 | 18 | 0.00085197 | High | 1792.02826 | [K].TPLNCPPGSLK.[V] |
| 746-756 | 3 | 3 | 0.00085197 | High | 1487.82111 | [K].TPLNCPPGSLK.[V] |
| 305-314 | 3 | 6 | 0.00085197 | High | 1716.98837 | [R].TLGMLMEGLK.[Q] |
| 820-830 | 3 | 13 | 0.00085197 | High | 1568.90075 | [K].TFLPIFINSVN.[-] |
| 305-314 | 3 | 3 | 0.00085197 | High | 1700.99345 | [R].TLGMLMEGLK.[Q] |
| 305-314 | 3 | 2 | 0.00085197 | High | 1412.78122 | [R].TLGMLMEGLK.[Q] |
| 305-314 | 3 | 5 | 0.00085197 | High | 1396.78631 | [R].TLGMLMEGLK.[Q] |
| 511-532 | 3 | 5 | 0.00085197 | High | 2738.34889 | [K].EQSPPLYCLFGPVPSPDVSGCK.[C] |
| 288-297 | 3 | 4 | 0.00085197 | High | 1335.79555 | [K].FGPVSGQVIK.[S] |
| 288-297 | 3 | 10 | 0.00085197 | High | 1640.0027 | [K].FGPVSGQVIK.[S] |
| 223-234 | 3 | 49 | 0.00085197 | High | 1964.07732 | [K].AATYFWPGSEVK.[I] |

| | | | | | | | | |
|---|---|---|---|---|---|---|---|---|
| | 223-234 | 3 | 2 | 0.00085197 | High | 1659.87017 | [K].AATYFWPGSEVK.[I] | | |
| | 370-381 | 3 | 5 | 0.00085197 | High | 2043.09303 | [K].DFYTFDSEAIVK.[K] | | |
| | 370-381 | 3 | 2 | 0.00085197 | High | 1738.88588 | [K].DFYTFDSEAIVK.[K] | | |
| | 533-545 | 3 | 8 | 0.00085197 | High | 1796.91318 | [K].CSSITDLEAVNQR.[L] | | |
| | 412-421 | 3 | 1 | 0.00085197 | High | 1811.07046 | [R].IDKVNLMVDR.[Q] | | |
| | 412-421 | 3 | 1 | 0.00085197 | High | 1506.86331 | [R].IDKVNLMVDR.[Q] | | |
| | 404-411 | 3 | 1 | 0.00085197 | High | 1288.74452 | [R].LHFANNIR.[I] | | |
| | 81-87 | 3 | 3 | 0.00165734 | High | 1569.75328 | [K].DCCTDYK.[S] | | |
| | 404-411 | 3 | 1 | 0.00246457 | High | 984.53737 | [R].LHFANNIR.[I] | | |
| | 403-411 | 3 | 1 | 0.00402361 | High | 1444.84563 | [K].RLHFANNIR.[I] | | |
| | 554-573 | 3 | 1 | 0.0058016 | High | 2611.33702 | [K].MQSEADNLPYGRPHVLQHSK.[Y] | | |
| | 298-304 | 3 | 2 | 0.0058016 | High | 1124.60531 | [K].SLQMADR.[T] | | |
| | 415-421 | 3 | 2 | 0.0058016 | High | 1150.65734 | [K].VNLMVDR.[Q] | | |
| P83234 | 158-168 | 1 | 9 | 0.00085197 | High | 2223.3569 | [K].IIVFLDYDKGK.[V] | Vespryn-21 | *O. hannah* |
| | 169-176 | 1 | 26 | 0.00085197 | High | 1514.9074 | [K].VIFDLDGK.[V] | | |
| | 169-176 | 1 | 3 | 0.00085197 | High | 1210.70025 | [K].VIFDLDGK.[V] | | |
| | 167-176 | 1 | 2 | 0.00085197 | High | 2004.23097 | [K].GKVIFDLDGK.[V] | | |
| D3TTC2 | 334-353 | 1 | 7 | 0.00085197 | High | 2569.22154 | [R].MVAITMAHEMGHNLGMNHDR.[G] | Zinc metalloproteinase-disintegrin-like atragin | *N. atra* |
| | 320-331 | 1 | 12 | 0.00085197 | High | 1587.82976 | [K].TSAAVVQDYSSR.[T] | | |
| | 272-281 | 1 | 15 | 0.00085197 | High | 1510.83373 | [R].ATLNLFGEWR.[E] | | |
| ENSNNAP00000015130.1 | 235-242 | 5 | 4 | 0.00085197 | High | 1620.90972 | [K].MVNYLNMK.[F] | Zinc metalloproteinase-disintegrin-like atragin | *N. atra* |
| | 291-303 | 5 | 9 | 0.00085197 | High | 2057.15227 | [R].NDNAQLLTGIDFK.[G] | | |
| | 291-303 | 5 | 9 | 0.00085197 | High | 1752.94513 | [R].NDNAQLLTGIDFK.[G] | | |
| | 290-303 | 5 | 2 | 0.00085197 | High | 2213.25338 | [K].RNDNAQLLTGIDFK.[G] | | |

| | | | | | | | | | |
|---|---|---|---|---|---|---|---|---|---|
| | 369-386 | 5 | 3 | 0.00085197 | High | 2459.1832 | [K].ISYEPLSEFSSCSVQEHR.[E] | | |
| | 320-330 | 5 | 6 | 0.00085197 | High | 1805.03003 | [R].TSVAVVQDYSK.[K] | | |
| | 320-330 | 5 | 3 | 0.00085197 | High | 1500.82289 | [R].TSVAVVQDYSK.[K] | | |
| | 235-242 | 5 | 1 | 0.0058016 | High | 1332.69749 | [K].MVNYLNMK.[F] | | |
| D5LMJ3 | 329-350 | 1 | 4 | 0.00085197 | High | 3052.57745 | [R].TILMASTMAHELGHNMGIHHDK.[A] | Zinc metalloproteinase-disintegrin-like atrase-A | *N. atra* |
| | 381-388 | 1 | 22 | 0.00085197 | High | 1391.76023 | [R].EHQEYLLR.[E] | | |
| | 457-465 | 1 | 2 | 0.00085197 | High | 1705.91871 | [K].FKGAETECR.[A] | | |
| | 466-491 | 1 | 11 | 0.00085197 | High | 3579.66455 | [R].AAKDDCDLPEFCTGQSAECPTDSLQR.[N] | | |
| | 355-380 | 1 | 2 | 0.00085197 | High | 3418.46553 | [R].CSHSPCIMSDTISDEPFYEFSSCSVR.[E] | | |
| | 355-380 | 1 | 1 | 0.00085197 | High | 3098.26347 | [R].CSHSPCIMSDTISDEPFYEFSSCSVR.[E] | | |
| | 560-570 | 1 | 15 | 0.00085197 | High | 2285.28137 | [K].CGTLYCTEIKK.[T] | | |
| | 560-570 | 1 | 4 | 0.00085197 | High | 1981.07422 | [K].CGTLYCTEIKK.[T] | | |
| | 560-569 | 1 | 7 | 0.00085197 | High | 1852.97926 | [K].CGTLYCTEIK.[K] | | |
| | 560-569 | 1 | 8 | 0.00085197 | High | 1548.77212 | [K].CGTLYCTEIK.[K] | | |
| | 508-532 | 1 | 4 | 0.00085197 | High | 3452.69664 | [K].CPTMENQCITLLGPNYTVGPAGCFK.[N] | | |
| | 508-532 | 1 | 6 | 0.00085197 | High | 3132.49458 | [K].CPTMENQCITLLGPNYTVGPAGCFK.[N] | | |
| | 355-380 | 1 | 1 | 0.00454195 | High | 3402.47061 | [R].CSHSPCIMSDTISDEPFYEFSSCSVR.[E] | | |
| | 457-465 | 1 | 1 | 0.00454195 | High | 1401.71156 | [K].FKGAETECR.[A] | | |
| ENSNNAP00000015250.1 | 525-544 | 2 | 3 | 0.00085197 | High | 2334.006 | [K].VSQDSCFTLNQIGQGCGFCR.[K] | Zinc metalloproteinase-disintegrin-like atrase-A | *N. atra* |
| | 525-544 | 2 | 5 | 0.00085197 | High | 2638.21314 | [K].VSQDSCFTLNQIGQGCGFCR.[K] | | |
| | 466-491 | 2 | 3 | 0.00085197 | High | 3625.68529 | [R].AAKDDCDLPEFCIGQSAECPTDSFQR.[N] | | |
| | 466-491 | 2 | 2 | 0.00085197 | High | 3321.47814 | [R].AAKDDCDLPEFCIGQSAECPTDSFQR.[N] | | |
| | 508-524 | 2 | 3 | 0.00085197 | High | 2510.37886 | [K].CPIMTNQCIALLGPSVK.[V] | | |

| | | | | | | | | | |
|---|---|---|---|---|---|---|---|---|---|
| | 508-524 | 2 | 3 | 0.00085197 | High | 2206.17172 | [K].CPIMTNQCIALLGPSVK.[V] | | |
| | 508-524 | 2 | 4 | 0.00085197 | High | 2526.37378 | [K].CPIMTNQCIALLGPSVK.[V] | | |
| ENSNNAP00000015377.1 | 466-491 | 1 | 17 | 0.00085197 | High | 3545.6802 | [R].AAKDDCDLPELCTGQSAECPTDSLQR.[N] | Zinc metalloproteinase-disintegrin-like atrase-A | *N. atra* |
| D6PXE8 | 291-309 | 2 | 4 | 0.00085197 | High | 2323.19615 | [R].NDNAQLLTGIDFNGNTVGR.[A] | Zinc metalloproteinase-disintegrin-like atrase-B | *N. atra* |
| | 291-309 | 2 | 5 | 0.00085197 | High | 2018.98901 | [R].NDNAQLLTGIDFNGNTVGR.[A] | | |
| | 290-309 | 2 | 2 | 0.00085197 | High | 2479.29726 | [K].RNDNAQLLTGIDFNGNTVGR.[A] | | |
| | 290-309 | 2 | 1 | 0.00085197 | High | 2175.09012 | [K].RNDNAQLLTGIDFNGNTVGR.[A] | | |
| | 332-353 | 1 | 3 | 0.00085197 | High | 3018.64388 | [R].ISLVASTMTHELGHNLGIHHDK.[A] | | |
| | 220-229 | 2 | 16 | 0.00085197 | High | 2138.2426 | [R].YYNNDKPAIK.[I] | | |
| | 451-476 | 2 | 33 | 0.00085197 | High | 3573.7115 | [R].VAKDDCDLPELCTGQSAECPTDSLQR.[N] | | |
| | 354-367 | 2 | 9 | 0.00085197 | High | 2244.18683 | [K].ASCICIPGPCIMLK.[K] | | |
| | 354-367 | 2 | 13 | 0.00085197 | High | 2228.19191 | [K].ASCICIPGPCIMLK.[K] | | |
| | 354-367 | 2 | 2 | 0.00085197 | High | 1923.98477 | [K].ASCICIPGPCIMLK.[K] | | |
| | 310-317 | 2 | 19 | 0.00085197 | High | 1519.8798 | [R].AYIGSLCK.[T] | | |
| | 262-275 | 2 | 7 | 0.00085197 | High | 2749.69685 | [K].DKFEVKPAASVTLK.[S] | | |
| | 248-263 | 2 | 2 | 0.00085197 | High | 2762.67488 | [K].IHIALIGLEIWSNKDK.[F] | | |
| | 220-229 | 2 | 3 | 0.00402361 | High | 1834.03545 | [R].YYNNDKPAIK.[I] | | |
| Q9PVK7 | 570-581 | 1 | 8 | 0.00085197 | High | 1904.99193 | [R].DPSYGMVEPGTK.[C] | Zinc metalloproteinase-disintegrin-like cobrin | *N. kaouthia* |
| | 570-581 | 1 | 6 | 0.00085197 | High | 1888.99702 | [R].DPSYGMVEPGTK.[C] | | |
| | 570-581 | 1 | 1 | 0.00085197 | High | 1584.78987 | [R].DPSYGMVEPGTK.[C] | | |
| | 260-269 | 1 | 1 | 0.00085197 | High | 1511.81774 | [K].ATLDLFGEWR.[E] | | |
| | 558-581 | 1 | 1 | 0.00923644 | High | 3324.59766 | [R].NSMICNCSISPRDPSYGMVEPGTK.[C] | | |

| | | | | | | | | | |
|---|---|---|---|---|---|---|---|---|---|
| A8QL59 | 567-580 | 1 | 1 | 0.00085197 | High | 2342.11358 | [R].LYCIDDSTEENSCK.[F] | Zinc metalloproteinase-disintegrin-like NaMP | *N. atra* |
| | 444-462 | 1 | 2 | 0.00085197 | High | 2909.36581 | [K].LKPEAQCASGACCEECQFR.[R] | | |
| Q10749 | 232-244 | 1 | 3 | 0.00085197 | High | 1907.96785 | [R].VYEMVNALNTMYR.[R] | Snake venom metalloproteinase-disintegrin-like mocarhagin | *N. mossambica* |
| | 232-244 | 1 | 3 | 0.00085197 | High | 1603.76071 | [R].VYEMVNALNTMYR.[R] | | |
| | 232-244 | 1 | 6 | 0.00085197 | High | 1923.96277 | [R].VYEMVNALNTMYR.[R] | | |
| | 321-330 | 1 | 2 | 0.00085197 | High | 1677.97794 | [K].SVAVVQDHSK.[S] | | |
| | 232-245 | 1 | 1 | 0.0058016 | High | 2080.06388 | [R].VYEMVNALNTMYRR.[L] | | |
| P82942 | 338-349 | 2 | 4 | 0.00085197 | High | 2233.25007 | [R].MEDGTKIPCAAK.[D] | Hemorrhagic metalloproteinase-disintegrin-like kaouthiagin | *N. kaouthia* |
| | 44210 | 1 | 1 | 0.00085197 | High | 2301.23304 | [-].TNTPEQDRYLQAEK.[Y] | | |
| | 29-38 | 1 | 3 | 0.00085197 | High | 2187.263 | [R].YYNYDKPAIK.[I] | | |
| | 191-198 | 1 | 4 | 0.00085197 | High | 1403.749 | [R].DYQEYLLR.[D] | | |
| | 338-349 | 2 | 1 | 0.00165734 | High | 1945.03784 | [R].MEDGTKIPCAAK.[D] | | |
| | 1-8 | 1 | 1 | 0.0058016 | High | 960.43811 | [-].TNTPEQDR.[Y] | | |

*Note*:Accession: from http://asia.ensembl.org/index.html or https://www.uniprot.org/
Confidence：High: q < 0.01, medium: 0.05–0.01
unique matching peptide sequences：Peptide sequences, including amino acids before and after in the peptide
Qvality q-value：Peptide q value
Proteins：Number of proteins containing the peptide
PSMs：Number of peptide matches contained in this peptide
Master Protein Accessions：Characteristic numbering of protein
Positions in accession：The position of this peptide in the protein
Theo. MH+ [Da]：Theoretical molecular weight of a peptide segment with one charge
Species: Matching by database http://asia.ensembl.org/index.html or https://www.uniprot.org/

**Table S2.** Peptide fraction separation liquid chromatography elution gradient table.

| Time (min) | Flow Rate (mL/min) | Mobile Phase A (%) | Mobile Phase B (%) |
|---|---|---|---|
| 0 | 1 | 97 | 3 |
| 10 | 1 | 95 | 5 |
| 30 | 1 | 80 | 20 |
| 48 | 1 | 60 | 40 |
| 50 | 1 | 50 | 50 |
| 53 | 1 | 30 | 70 |
| 54 | 1 | 0 | 100 |

**Table S3.** Liquid chromatography elution gradient table.

| Time (min) | Flow Rate (nL/min) | Mobile Phase A (%) | Mobile Phase A (%) |
|---|---|---|---|
| 0 | 600 | 94 | 6 |
| 2 | 600 | 85 | 15 |
| 78.5 | 600 | 60 | 40 |
| 80.5 | 600 | 50 | 50 |
| 81.5 | 600 | 45 | 55 |
| 90 | 600 | 0 | 100 |
